# Comparison of Adjuvant Potency of Alum, AddaVax, and ISA 71 VG on the Seasonal Split Influenza Vaccine in Mice

**DOI:** 10.3390/microorganisms13112542

**Published:** 2025-11-06

**Authors:** Li Wu, Rui Yang, Huimin Wu, Beibei Yang, Xin Zhang, Yingying Tao, Xing Wu, Shaozhen Li, Jianhong Shu, Yulong He, Huapeng Feng

**Affiliations:** 1 Department of Biology, College of Life Sciences, China Jiliang University, Hangzhou 310018, China; y18867136783@outlook.com; 2 Department of Biopharmacy, College of Life Sciences and Medicine, Zhejiang Sci-Tech University, Hangzhou 310018, China; whmtse@foxmail.com (H.W.); yangbeibeiybb@foxmail.com (B.Y.); zhangxin20000112@gmail.com (X.Z.); taoyingying20011009@gmail.com (Y.T.); xingtuotuo@foxmail.com (X.W.); lsz2024@yeah.net (S.L.); shujianhong@zstu.edu.cn (J.S.); heyl79@zstu.edu.cn (Y.H.)

**Keywords:** adjuvant effect, three adjuvants, split influenza vaccine, mouse model

## Abstract

Influenza is a highly contagious disease and is transmitted by the upper respiratory tract. Vaccination is an effective strategy to prevent and control seasonal influenza. The current predominant split-inactivated influenza vaccine presents a high safety profile but has weak immunogenicity. The addition of adjuvants is one method to optimize the immunogenicity of the seasonal influenza vaccine. In this study, we compared the effect of aluminum (Alum), MF59-like adjuvant AddaVax, and ISA71 VG adjuvants for the seasonal split influenza vaccine in a mouse model based on the induction of influenza-virus-specific antibody levels, body weight changes, and survival rates after lethal challenge. Two very low and sub-optimal HA doses, 0.003 µg and 0.01 µg, representing the calculated amount of HA from the A/California/07/2009 (H1N1) strain only per mouse dose, were selected and used in this study. The 0.003 µg antigen (Ag) plus AddaVax showed the best adjuvant effect among these three adjuvants. The 0.01 µg Ag plus ISA 71 VG induced the highest total IgG, IgG1, and IgG2a. Both the 0.003 µg and 0.01 µg Ag plus AddaVax protected all the immunized mice from the lethal challenge, and Alum exhibited the protective potency intermediate between that of the AddaVax and ISA 71VG. The 0.01 µg Ag plus one of these three adjuvants could enhance the efficacy of the split influenza vaccine against lethal challenge. Therefore, AddaVax is the first candidate for the further development of the adjuvanted split seasonal influenza vaccine among these three adjuvants. These initial findings offer valuable guidance for selecting promising adjuvanted influenza vaccine formulations.

## 1. Introduction

Influenza is a highly contagious disease caused by influenza virus infection. Seasonal influenza causes epidemics each year worldwide, resulting in severe disease, especially in young children and the elderly, posing a severe threat to public health all over the world [1]. The most recent influenza pandemic was caused by pdmH1N1 2009 influenza virus, which infected approximately 10~20% humans [2]. H7N9 avian influenza viruses were pandemic in poultry from 2013 to 2017 and caused 1568 human cases with 616 deaths, with a fatality rate of up to 39% in China [3,4]. From March 2024, the highly pathogenic H5N1 avian influenza viruses belonging to Clade 2.3.4.4b became endemic in dairy cattle in the USA and have infected 67 humans with 1 fatality [5]. Vaccination is the most effective strategy to prevent and control influenza disease. There are three types of influenza vaccines available for humans, including the inactivated influenza vaccine, live attenuated influenza vaccine, and subunit influenza vaccine against the H1N1, H3N2, and B/Victoria with or without B/Yamagata influenza viruses [6]. The FDA recommends excluding the B/Yamagata since this virus almost did not circulate after the COVID-19 pandemic in the 2024–2025 season influenza vaccine [7,8]. The main type of influenza vaccine used in China is the inactivated influenza vaccine because of its good safety profile. The effectiveness of the inactivated influenza vaccine varies from 19% to 60% up to the matched rate between the vaccine strain and circulating strain [9,10]. The effectiveness of the current inactivated influenza vaccine is low to moderate from year to year. The efficacy of the seasonal influenza vaccine needs to be further improved.

The addition of adjuvants is one effective method to improve the efficacy of the influenza vaccine. The adjuvants used in licensed commercial influenza vaccines are Alum, MF59 (the commercial counterpart of AddaVax), AS03, AF03, virosomes, and heat-labile enterotoxin (LT). However, some, like LT and AF03, are no longer in use due to safety concerns or lack of marketing [11]. Aluminum (Alum) is the first adjuvant used for the human vaccine since the 1920s and has been used for multiple human vaccines until now, including the SARS-CoV-2 vaccine, hepatitis B vaccines, HPV vaccine, DTaP vaccines, the pneumococcal conjugate vaccine, and so on [12,13]. Alum has a good safety profile and induction of a strong Th2-biased immune response but lacks the ability to induce the Th1-biased immune response. In addition, alum has the potential ability to induce an allergy, since alum could increase the level of IgE antibodies [14,15]. The MF59-like adjuvant AddaVax is a squalene-based oil-in-water emulsion adjuvant. It could induce a balanced Th1 and Th2 immune response to enhance humoral and cellular immunity [16]. AddaVax is similar to the MF59 adjuvant, which has been licensed for use in some influenza vaccines in Europe and by the FDA, especially for the elderly [17]. Currently, AddaVax is being used in the research and development of vaccines for various diseases, including influenza, SARS-CoV-2, yellow fever, and ovarian cancer, some of which have entered clinical trials with an acceptable safety profile [18,19]. ISA 71 VG is a commercial mineral oil-based water-in-oil adjuvant, which is mainly used to enhance the cellular immune response and is regarded as safe and is approved for the veterinary market under European Council Regulations [20]. ISA 71 VG has shown good adjuvant activity in poultry vaccines against pathogens such as H5N1 and H9N2 avian influenza viruses and Newcastle disease virus [21]. Although the effects of MF59, ISA 70 VG, and nano-aluminum hydroxide adjuvants on avian influenza plus Newcastle disease vaccine have been reported in chickens, the potency comparison of Alum, AddaVax, and ISA 71 VG on seasonal split influenza vaccine in mammals remains unclear. The aim of this study was to compare the adjuvant effect of Alum, AddaVax, and ISA 71 VG on the seasonal split influenza vaccine in a mouse model to facilitate the development of adjuvanted influenza vaccines.

## 2. Materials and Methods

### 2.1. Ethics Statement

All experiments with mice were performed following the Regulations for Animal Care and the Guidelines for Proper Conduct of Animal Experiments by the Zhejiang Sci-Tech University (ZSTU). All of the experiments were approved by the Experimental Animal Welfare Ethics Committee of the College of Life Sciences, Zhejiang Sci-Tech University (ZSTU) (protocol code 20220304-16, approved date: 4 March 2022).

### 2.2. Cells and Viruses

Madin–Darby canine kidney cells (MDCK, Cat. No: CCL-34) were purchased from American Type Culture Collection (ATCC) and were cultured in Dulbecco’s Modified Eagle Medium (DMEM) (Gibco, Waltham, MA, USA) containing 5% fetal bovine serum (FBS) with 1% penicillin-streptomycin solution (Gibco). Human embryonic kidney epithelial cells (293T, Cat No. CRL-3216) were obtained from ATCC and cultured in DMEM containing 10% FBS with 1% penicillin-streptomycin solution (Gibco) in a 37 °C with 5% CO_2_ incubator.

A/California/07/09 (CA07, H1N1) was amplified in MDCK cells at 33 °C and was used to coat the plates for the ELISA. The mouse-adapted A/California/04/09 (MA-CA04, H1N1) virus used for the lethal challenge was generated using a reverse genetics system. Specifically, cloned cDNA plasmids encoding the A/California/04/09 (CA04, H1N1) genome, engineered to contain specific mouse-adapting mutations, were transfected into 293T cells to rescue the virus. The rescued and pure MA-CA04 virus was then propagated in MDCK cells and stored at −80 °C until use as previously described [22,23]. The HA protein of CA07 contains four amino acid substitutions (D127E, K142N, A197T, D222G) relative to the HA protein of MA-CA04. Both strains were antigenically similar but not identical.

### 2.3. Preparation of Influenza Vaccines and Adjuvants

The inactivated split-virion influenza vaccine from Denka Seiken (Tokyo, Japan) for the 2014–2015 season was used as the antigen (Ag). This quadrivalent vaccine contained hemagglutinin (HA) proteins from four strains: A/California/07/2009 (H1N1), A/Hong Kong/4801/2014 (H3N2), B/Phuket/3073/2013 (Yamagata lineage), and B/Texas/2/2013 (Victoria lineage). Each original vial contained 30 µg of HA protein from each virus. For the mouse study, this stock was serially diluted to prepare the tested doses. The 0.003 µg and 0.01 µg doses represent the calculated amount of HA from the A/California/07/2009 (H1N1) strain only per mouse dose.

Alum-based adjuvant Alhydrogel^®^ adjuvant 2% (InvivoGen, San Diego, CA, USA) was mixed with the corresponding antigen with equal volume (antigen: alum = 1:1 (*v*/*v*), approximately equal to 500 μg of alum per dose) and incubated the mixture for 3 h at 4 °C.

AddaVax adjuvant (InvivoGen, San Diego, CA, USA) was also mixed with diluted antigen with equal volume-antigen: Addavax = 1:1 (*v*/*v*), approximately equal to 50 μL of AddaVax (about 49.8 mg) per dose as recommended by the manufacturer and incubated with each other for 3 h at 4 °C. Montanide^TM^ ISA 71 VG adjuvant (Seppic, Paris, France) was mixed with antigen according to the 70% volume of adjuvant and the 30% volume of antigen solution (antigen: ISA 71 VG = 3:7 (*w*/*w*), approximately equal to 70 mg of ISA71VG/dose) as recommended by the manufacturer and incubated with gentle rotation at room temperature for 30 min.

### 2.4. Immunization and Challenge with MA-CA04

Five-week-old female BALB/c mice were purchased from Shanghai SLAC Laboratory Animal Co., Ltd. (Shanghai, China). The mice were randomly divided into different groups, four mice per group. After a one week adaptation, the mice were immunized intramuscularly with 100 μL respective influenza vaccine formulation at the two hind legs (50 µL per leg). The mice were injected twice at two-week intervals. Two weeks after the secondary immunization, blood samples were collected from the submandibular vein of the immunized mice with a 5 mm Goldenrod Animal Lancet (Braintree Scientific, Braintree, MA, USA), and the sera were isolated for antibody level detection.

After the blood sample collection, 10 MLD_50_ (fifty percent mouse lethal dose) MA-CA04 virus in 50 μL volume was used to challenge the immunized mice under dry ice-induced anesthesia. Then the body weight and survival were detected each day for 14 days. If the reduced body weight of the mice was equal to or more than 25% of the original body weight, the mice would be euthanized and calculated as death according to the regulations of animal welfare.

### 2.5. ELISA Detection for the Antibody Level in Sera

The ELISA plate was coated with purified, inactivated CA07 virus (6 μg total viral protein/mL) as the antigen, with 50 μL per well in PBS, and incubated at 4 °C overnight. The coating antigen was then removed, and the plates were washed once with PBS plus 0.5% Tween 20 (PBS-T). Then the plates were blocked by using the Blocking One (Nakalai Tesque, Kyoto, Japan) for 1 h at room temperature (RT). The diluted sera collected from the immunized mice were added to each well and incubated for 1 h at RT. The diluted sera were removed. The wells of the plates were washed four times with PBS-T, and the peroxidase-labeled goat anti-mouse IgG (γ) antibodies (5220-0339, SeraCare, Milford, MA, USA) for detecting total IgG, horseradish peroxidase-conjugated anti-mouse IgG1 or IgG2a antibodies (Southern Biotech, Birmingham, AL, USA), were added at 1 × 2000 dilution for 1 h incubation. After 1 h incubation, the plates were washed four times with PBS-T. 2,2′-Azinobis[3-ethylbenzothiazoline-6-sulfonic acid]-diammonium salt (ABTS) substrate solution was added into each well and incubated for 15 min at RT. The results were detected by a microplate reader at 405 nm wavelength. The endpoint titer corresponded to the reciprocal of the maximal serum dilution yielding an optical density > 0.1 at 405 nm following negative serum control correction [24].

### 2.6. Statistical Analysis

Data were analyzed using the Mann–Whitney U test with GraphPad Prism 6.0 (GraphPad Software Inc., San Diego, CA, USA). *p* < 0.05 was considered to be a significant difference.

## 3. Results

### 3.1. Alum, AddaVax, and ISA71 VG Enhance the Immunogenicity of the Split Seasonal Influenza Vaccine

**Figure 1:**
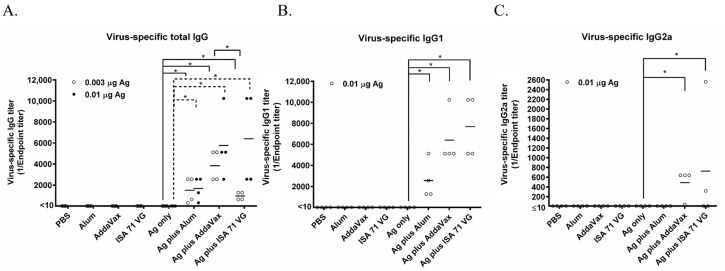
Virus-specific antibody titers elicited by Alum, AddaVax, or ISA 71 VG plus Ag in mice. Six-week-old BALB/c mice (*n* = 4 per group) were immunized with the Ag plus Alum, AddaVax, or ISA 71 VG adjuvant for the first time at day 0. After two weeks, these mice were vaccinated by using the same vaccine formulations a second time. The sera were isolated from the blood samples collected at day 14 after the second vaccination. ELISA was used to detect the virus-specific total IgG (**A**), IgG1 (**B**), and IgG2a (**C**) antibodies with purified CA07 viral particles as the coating antigen. * *p* < 0.05.

PBS, Alum, AddaVax, and ISA71 VG did not induce the generation of influenza virus-specific antibodies. The 0.003 μg and 0.01 μg Ag (which were calculated based on CA07 HA) vaccines alone also did not induce the production of influenza virus-specific antibodies. Both 0.003 μg HA plus Alum and 0.01 μg Ag plus Alum significantly enhanced the antibody level compared to the corresponding Ag alone group; 0.01 μg Ag plus Alum induced a slightly higher total IgG antibody titer than that of 0.003 μg Ag plus Alum; both 0.003 μg Ag plus AddaVax and 0.01 μg Ag plus AddaVax significantly enhanced the influenza virus-specific antibody titers (Figure 1A). Among the three 0.003 μg Ag plus adjuvant groups, 0.003 μg Ag plus AddaVax elicited the highest total IgG antibody level. The 0.003 μg Ag plus ISA 71 VG induced a lower total IgG antibody titer than that elicited by 0.003 μg Ag plus AddaVax (Figure 1A). Therefore, AddaVax exhibits the highest adjuvant effect for enhancing the immunogenicity of the split-inactivated influenza vaccine.

To detect the classification of these three adjuvants-induced immune responses, we used the ELISA to determine the titers of the influenza virus-specific IgG1 and IgG2a antibodies. The results showed that the IgG1 antibodies stimulated by 0.01 µg Ag + Alum, 0.01 µg Ag + AddaVax, or 0.01 µg Ag + ISA 71 VG significantly increased compared to that induced by Ag alone (Figure 1B). The IgG2a response was induced by 0.01 µg Ag + AddaVax or 0.01 µg Ag + ISA 71 VG, while it is not elicited by 0.01 µg Ag + Alum or Ag alone (Figure 1C). Vaccine-induced IgG subclass patterns correlate with adaptive immune responses: murine IgG1 production signifies a Th2-polarized response, while IgG2a dominance suggests a Th1-oriented response. These findings establish that AddaVax and ISA 71 VG prompt both Th1 and Th2 immune responses, whereas Alum elicits solely a Th2 response.

### 3.2. Alum, AddaVax, and ISA 71 VG Improve the Efficacy of the Split Influenza Vaccine Against Lethal Challenge

**Figure 2:**
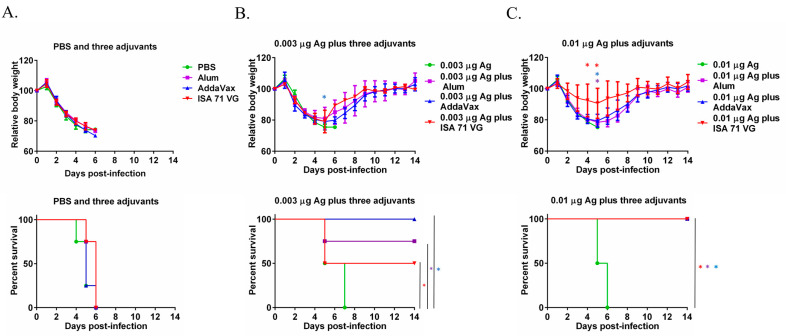
The efficacy of Ag plus Alum, AddaVax, or ISA 71 VG adjuvants against lethal challenge. Six-week-old mice (*n* = 4 per group) were immunized with Ag with or without Alum, AddaVax, or ISA 71 VG twice and were challenged with 10 MLD_50_ MA-CA04 virus on day 14 after the second immunization. (**A**) Body weight change and survival rate of the mice injected with PBS, Alum, AddaVax, or ISA 71 VG only post-challenge. (**B**) Body weight change and survival rate of the mice immunized with 0.003 μg Ag plus Alum, AddaVax, or ISA 71 VG post-challenge. (**C**) Body weight change and survival rate of the mice immunized with 0.01 μg Ag plus Alum, AddaVax, or ISA 71 VG post-challenge. * *p* < 0.05. Purple asterisks indicate statistically significant differences (*p* < 0.05 in the comparison between the Ag plus Alum group and the Ag alone group; blue asterisks indicate statistically significant differences (*p* < 0.05) in the comparison between the Ag plus AddaVax group with the Ag alone group; red asterisks indicate statistically significant differences (*p* < 0.05) in the comparison between the Ag plus ISA 71 VG group and the Ag alone group.

On day 14 after the secondary immunization, all the mice were challenged with 10 MLD50 MA-CA04 virus by intranasal inoculation. As shown in Figure 2A, the mice injected with PBS, Alum, AddaVax, or ISA 71 VG alone were all dead by day 6 post-challenge. For the 0.003 μg Ag alone group, the mice were all dead on day 7 post-challenge. All the mice immunized with 0.003 μg Ag plus AddaVax survived after lethal challenge. The survival rate was 75% after the challenge in the 0.003 μg Ag plus Alum group and 50% of the mice immunized with 0.003 μg Ag plus ISA 71 VG survived post-challenge (Figure 2B). The mice immunized with the 0.01 μg Ag were all dead on day 6 post-challenge, which is consistent with the antibody titers shown in Figure 1A. 0.01 μg Ag plus Alum, AddaVax, and ISA 71 VG provide complete protection from the 10 MLD_50_ MA-CA04 challenge. The mice immunized with 0.01 μg Ag plus ISA 71 VG had the lowest body weight change among these three adjuvant groups. The above results suggest that AddaVax exhibited the best overall adjuvant effect while ISA 71 VG showed the least body weight reduction (indicating strong protection) when combined with 0.01 μg Ag influenza vaccine (Figure 2C).

## 4. Discussion

Influenza poses a severe threat to public health and the economy. Vaccination is the most effective strategy to prevent and control influenza. Since the influenza virus evolves rapidly, the development of a more effective influenza vaccine is urgently needed. The addition of adjuvants is one of the effective methods to improve the efficacy of the influenza vaccine, especially for the inactivated split or subunit influenza vaccine. In this study, we selected three different types of adjuvants, evaluated and compared their adjuvant effect on the inactivated split influenza vaccine, including the aqueous adjuvant Alum, oil-in-water-based AddaVax, and water-in-oil-based ISA 71 VG.

The immunogenicity of the vaccine is an important and easily determined parameter in animal models and clinical trials. The antibody titers are commonly used for evaluating the immunogenicity of the influenza vaccine. Our results showed that AddaVax is superior to ISA 71 VG and that ISA 71 VG is better than Alum (Figure 1). Bai et al. demonstrate that the MF59-like adjuvant AddaVax induced higher neutralization antibody titers than that of Alum for the severe acute respiratory syndrome coronavirus 2 (SARS-CoV-2) vaccines in aged mice [25]. In adults, MF59 increases the HI antibody titers up to 2–5 fold compared to that of Alum for the H5N1 influenza subunit vaccine [26]. The AddaVax-adjuvanted H5N8 subtype inactivated influenza vaccine stimulates a stronger immune response than the Alum-based formulation in mice [27]. These reports are consistent with the results of this study (Figure 1). ISA 71 VG is a mineral-based water-in-oil type adjuvant and is usually used for poultry or cow vaccines. ISA 71 VG is superior to Freund’s adjuvant in the vaccines against S. aureus infection in mice [20]. The ISA 71 VG-like adjuvant ISA 70 VG stimulates higher antibody titers than that of nano-Alum in chickens, and our result showed that ISA 71 VG elicits higher antibody titers than that of Alum when in combination with 0.01 μg Ag vaccine in mice (Figure 1), which is in line with our results. However, while one study found that the induction activity of AddaVax was weaker than that of nano-Alum for an avian influenza and Newcastle disease bivalent inactivated vaccines in chickens, our result showed that the Ag plus AddaVax induced a higher antibody level than the Ag plus Alum in mice (Figure 1) [28]. The controversial conclusion may be attributed to the different animal models in these two studies; the adjuvant effect of the water-in-oil type adjuvant may be better than that of the oil-in-water type in chickens, which have an immune system different from that of mammals. Meanwhile, ISA 71 VG improved the humoral immunity for the H5N1, H9N2 avian influenza, and Newcastle disease trivalent inactivated vaccines in chicken [21]. ISA 71 VG was an effective adjuvant for the prefusion F subunit vaccine against respiratory syncytial virus in bovine [29]. Therefore, we can know that the adjuvant effect of MF59 or AddaVax is better than that of Alum in the mammalian models, while it is weaker in poultry. ISA 71 VG showed good adjuvant activity in both poultry and mammalian animal models. Therefore, either oil-in-water or water-in-oil adjuvants can be chosen for mammalian models, while water-in-oil adjuvants may be preferable for poultry.

In this study, we selected two doses of antigens for our evaluation: 0.003 µg and 0.01 µg Ag. Based on the antibody level and protective efficacy, we know that 0.01 µg Ag plus all three adjuvants is generally better than 0.003 µg plus adjuvants (Figure 1 and Figure 2). However, both 0.003 µg and 0.01 µg Ag per dose did not elicit the production of virus-specific antibodies (Figure 1). Therefore, we know that both 0.003 µg and 0.01 µg Ag per dose are sub-optimal doses for the influenza vaccine. And 0.01 µg Ag per dose seems to be the optimum dose of Ag for our comparison.

Protection rate by lethal challenge is one gold standard to evaluate the efficacy of vaccines. The Alum, AddaVax, and ISA 71 VG protected all the immunized mice against lethal challenge when combined with 0.01 μg Ag (Figure 2C). When combined with 0.003 μg Ag, 100% of mice in the Ag + AddaVax group survived after the challenge, 75% of mice immunized with Ag + Alum were alive post-challenge, while only 50% of mice in the Ag + ISA 71 VG group survived after the lethal challenge (Figure 2B). AddaVax showed better protective efficacy than Alum for inactivated Yellow Fever 17DD vaccine candidate in mice, which is consistent with our results [30]. Alum exhibits the adjuvant effect mainly by activating the NLPR3 inflammasome to promote the release of IL-1β and IL-18 cytokines and further boost the Th2 antibody immune response [31], which is consistent with our finding that Alum induced high IgG1 antibody levels while Alum did not elicit the production of IgG2a antibodies (Figure 1B,C). AddaVax and MF59 are similar; MF59 is composed of 3.9% squalene with weight/volume (*w/v*) ratio, 0.47% Tween 80 with *w*/*v*, 0.47% Span 85 (*w*/*v*), and 10 mM citrate buffer with pH 6.0. MF59 and MF59-like adjuvants exert adjuvant activity by stimulating the release of chemokines CCL2, CCL4, CCL5, and CXCL8, which recruit immune cells such as macrophages, monocytes, and granulocytes to the injection site, generating a local immunocompetent environment. These recruited cells then take up the antigen and MF59 or AddaVax to deliver them to the lymph node to activate the adaptive immune response [18,32]. MF59 and AddaVax induced robust antigen-specific antibody and CD8+ T cell immune response in draining lymph nodes by activating the RIPK3-kinase dependent signal pathway in mice, while alum did not induce the activation of RIPK3-kinase dependent signaling [33]. MF59 could also activate the T follicular helper cells (Tfh) to promote the amplification of the germinal center (GC) B cell response [34]. In this study, Ag plus AddaVax stimulated the generation of both IgG1 and IgG2a antibodies, indicating it induced Th1 and Th2 immune responses (Figure 1B,C). Thus, the good efficacy of AddaVax in seasonal influenza vaccine is due to its ability to induce both humoral and cellular immunity. ISA 71 VG is a water-in-oil adjuvant and is composed of light mineral oil, mannitol, and oleic acid emulsifier [28]. ISA 71 VG could enhance the Th1 immune response and the production of IgG2 antibodies. Both Th1 immune responses and IgG2 antibodies are important for the prevention of infectious diseases; Th1 immune response induces the IFN-γ to combat the intracellular pathogens, while IgG2 could fight the extracellular pathogens [35]. One study demonstrated that ISA 71 VG showed better protective efficacy than Alum when combining with recombinant H1N1 HA vaccine against swine influenza virus in a mouse model [36]. In this study, the mice immunized with 0.01 μg Ag plus ISA 71 VG exhibited the least body weight reduction among all the groups (Figure 2C). Our result is similar to the previous study. This result may be attributed to both the specific Th1 immune response and IgG2 antibodies in mice. In addition, the differential survival rates observed with varying antigen doses underscore the critical interplay between adjuvant mechanism and antigen availability. While Alum provided superior protection (75% survival) to ISA 71 VG (50%) at the 0.003 µg dose despite comparable total IgG levels (Figure 2A,C), this can be attributed to their distinct immune polarizations. Alum’s strong Th2-biased response, characterized by high IgG1, may efficiently generate antibodies even with minimal antigen, sufficient for partial protection. Conversely, the potent Th1-skewed immunity induced by the water-in-oil ISA 71 VG adjuvant, which is crucial for cellular responses, likely requires a higher antigenic stimulus to become fully protective. This explains why ISA 71 VG’s efficacy surged to 100% survival at the 0.01 µg dose, where the antigen was sufficient to elicit a robust and balanced response. Thus, the nature and quality of the immune response, dictated by the adjuvant’s mechanism, are as critical as its magnitude, and their efficacy can be highly dependent on Ag dose.

In conclusion, we compared the adjuvant activity of Alum, AddaVax, and ISA 71 VG on the seasonal split influenza vaccine in mammalian model. In general, all these three adjuvants could improve the immunogenicity of the influenza vaccine. AddaVax elicits higher antibody titers compared to Alum and ISA 71 VG when combined with low-dose Ag, AddaVax and ISA 71 VG induce high and comparable antibody titers when combined with high dose Ag, and Alum induces less antibody production. AddaVax provides complete protection at both low-dose Ag and high-dose Ag against lethal challenge in mice. Alum and ISA 71 VG provide partial protection when combined with low-dose Ag and both of them provide complete protection with high-dose Ag for lethal challenge. In the future, we could consider combining Alum, AddaVax, or ISA 71 VG with other compounds to further enhance the efficacy and durability of influenza vaccines. For example, AddaVax plus CpG, Alum plus Poly (I: C), and ISA 71 VG plus QS21. Our results serve as a reference for selecting appropriate adjuvants for influenza vaccines, guiding the development of more effective adjuvanted influenza vaccines.

## Data Availability

The original contributions presented in this study are included in the article. Further inquiries can be directed to the corresponding authors.
